# Association between adolescent idiopathic scoliosis prevalence and age at menarche in different geographic latitudes

**DOI:** 10.1186/1748-7161-1-9

**Published:** 2006-05-23

**Authors:** Theodoros B Grivas, Elias Vasiliadis, Vasilios Mouzakis, Constantinos Mihas, Georgios Koufopoulos

**Affiliations:** 1 Orthopaedic Department, "Thriasio" General Hospital, G. Gennimata Av. 19600, Magoula, Attica, Greece

## Abstract

**Background:**

Age at menarche is considered a reliable prognostic factor for idiopathic scoliosis and varies in different geographic latitudes. Adolescent idiopathic scoliosis prevalence has also been reported to be different in various latitudes and demonstrates higher values in northern countries. A study on epidemiological reports from the literature was conducted to investigate a possible association between prevalence of adolescent idiopathic scoliosis and age at menarche among normal girls in various geographic latitudes. An attempt is also made to implicate a possible role of melatonin in the above association.

**Material-methods:**

20 peer-reviewed published papers reporting adolescent idiopathic scoliosis prevalence and 33 peer-reviewed papers reporting age at menarche in normal girls from most geographic areas of the northern hemisphere were retrieved from the literature. The geographic latitude of each centre where a particular study was originated was documented. The statistical analysis included regression of the adolescent idiopathic scoliosis prevalence and age at menarche by latitude.

**Results:**

The regression of prevalence of adolescent idiopathic scoliosis and age at menarche by latitude is statistically significant (p < 0.001) and are following a parallel declining course of their regression curves, especially in latitudes northern than 25 degrees.

**Conclusion:**

Late age at menarche is parallel with higher prevalence of adolescent idiopathic scoliosis. Pubarche appears later in girls that live in northern latitudes and thus prolongs the period of spine vulnerability while other pre-existing or aetiological factors are contributing to the development of adolescent idiopathic scoliosis. A possible role of geography in the pathogenesis of idiopathic scoliosis is discussed, as it appears that latitude which differentiates the sunlight influences melatonin secretion and modifies age at menarche, which is associated to the prevalence of idiopathic scoliosis.

## Background

A wide range of Adolescent Idiopathic Scoliosis (AIS) prevalence in different countries is demonstrated by the various reports in the literature. (1–20) The significance of this specific observation may not be obvious but its evaluation is important because it could be related to a possible contributory factor of AIS pathogenesis.

In studying variations on the rate of sexual development across the world, a similar observation is recorded for the age at menarche, as well. (21–53) The influence of the geography of a specific region on human biology is determined by socioeconomic and environmental factors such as temperature, humidity and lighting that are transferred and expressed in human cells by specific mediators. (54) Age at menarche is definitely a biologic event and is considered a reliable prognostic factor of AIS. (55, 56)

The aim of this report is the study of IS prevalence and age at menarche as it is reported on published papers from different countries in various geographic latitudes and the investigation of a possible association between them that may reveal their possible role on AIS pathogenesis.

## Methods and material

The inclusion criteria for the epidemiological studies were clearly defined before performing a search of the literature on scoliosis prevalence and on age at menarche by browsing the Medline database. The geographic latitude of each centre where a particular study was originated was documented. The included studies cover the whole spectrum of geographic latitudes in the northern hemisphere.

### The scoliosis prevalence

A paper was considered eligible for inclusion when the study was reporting the prevalence of AIS among normal girls, was age matched, involving girls between 10 and 14 years old, the curves were detected through screening programs and a cut-off point of 10 degrees of Cobb angle was used for AIS definition. Twenty peer-reviewed papers met those criteria and were included in the study [1-20].

### The age at menarche

Thirty three peer-reviewed papers reporting on age at menarche in normal girls at certain geographic regions were found and were included in the study [21-53]. All the included papers are reporting the age at menarche of normal girls and not of specific groups.

### The statistical analysis

The prevalence of idiopathic scoliosis and age at menarche were treated as the dependent variables in a linear regression forward modeling procedure. The geographic latitude of each location where the study had taken place was the candidate independent variable. Because of the different sample size of each study, frequency weights were used in the regression model, controlling for the impact of each latitude value, according to their sample size. The *F*-test of significance of overall regression and Type I partial *F*-test were calculated at a significance level less than 0.05, testing for the significance of overall regression and for each variable added during the modeling. A graphical analysis of the residuals of the regression was performed in order to detect potential problems with the model. Data were analysed using STATA™ (Version 8.0, Stata Corporation, College station, TX 77845, 800-782-8272).

## Results

### The scoliosis prevalence

**Figure 1:**
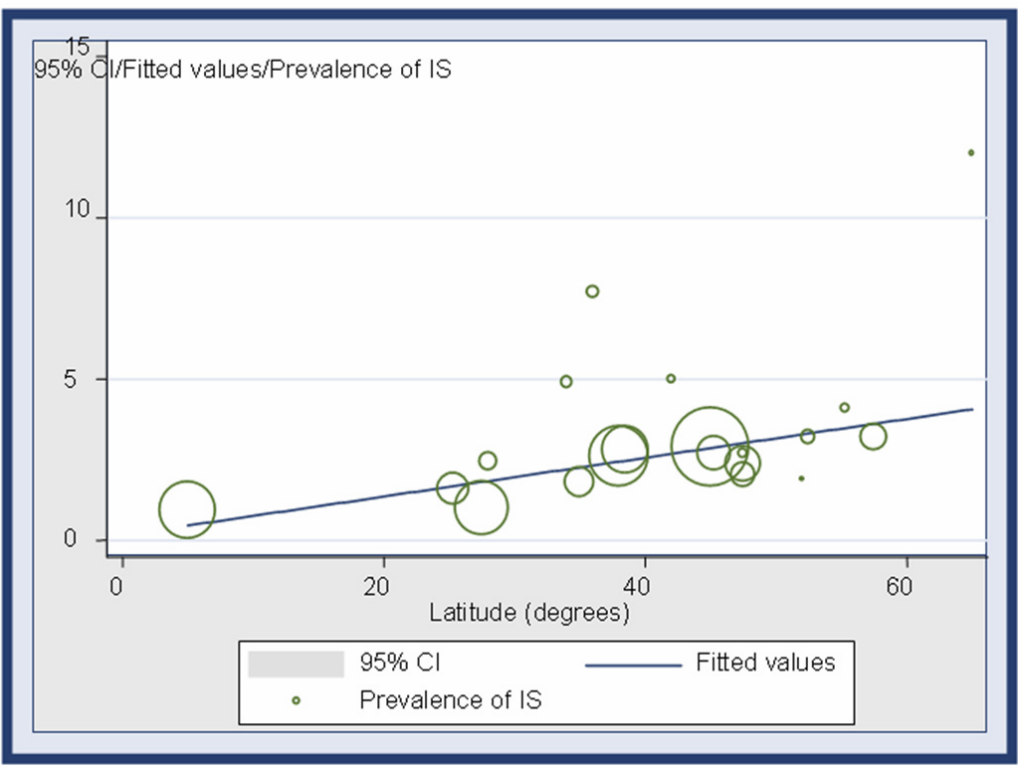
Frequency weighted linear regression plot. The circle size represents the sample size of each study.

The reported prevalence of AIS in the literature increases in the northern geographic latitudes and decreases as the latitude is approaching the equator (Table 1, Figure 1).

**Table 1:** Demonstrates data on AIS prevalence (%) according to the city or geographical periphery that the epidemiological study was performed, the size of the sample of the examined girls, the geographic latitude in degrees and the author and the year of publication.

**City or Geographical Periphery**	**Geographic Latitude in degrees**	**No of girls screened**	**Prevalence of IS (%)**	**Author and year of publication**
Helsinki (Finland)	65,00	401	**12,0**	Nissinen M et al 1993 [1]
Malmo (Sweden)	57,50	8469	**3,21**	Willner S & Uden A 1982 [2]
Esbjerg (Denmark)	55,30	1000	**4,10**	Laulund T et al 1982 [3]
Oxford (UK)	52,50	2613	**3,20**	Dickson RA et al 1983 [4]
Nottingham(UK)	52,00	321	**1,90**	Burwell RG et al 1983 [5]
Quebec (Canada)	47,50	14701	**2,37**	Morais Tet al 1985 [6]
Rochester (Minn, USA)	47,50	1212	**2,70**	Yawn BP et al 1999 [7]
Wisconsin (USA)	47,50	7462	**2,03**	Gore DR et al 1981 [8]
Slovenia	45,00	70200	**2,89**	Plenicar-Cucek M et al 1995 [9]
Montreal (Canada)	45,30	13500	**2,70**	Rogala EJ et al 1978 [10]
Cape Cod (USA)	42,00	928	**5,00**	Strayer LM et al, 1973 [11]
Delaware (USA)	38,50	25550	**2,80**	Shands AR et al 1955 [12]
Epirus (Greece)	38,00	40962	**2,60**	Soucacos PN et al 1997 [13]
California (USA)	36,00	1940	**7,70**	Brooks HL et al 1975 [14]
Crete (Greece)	35,00	10278	**1,80**	Koukourakis I et al 1997 [15]
Wakayaka (Japan)	34,00	1702	**4,90**	Sugita K 2000 [16]
Hu Guan (China)	25,30	12000	**1,60**	Ma X et al 1995 [17]
Changsha (China)	28,00	3963	**2,45**	Pin LH et al 1985 [18]
Taipei (Taiwan)	27,50	33596	**1,00**	Huang SC 1997 [19]
Singapoure	5,00	37141	**0,93**	Wong HK et al 2005 [20]

The final regression model is shown in Table 2. The inclusion of the quadratic term of latitude contributed to the explanatory power of the model according to partial *F *test and overall *F *test. According to the modelling of the data, a significant positive association between prevalence of IS and latitude was found (overall F p-value < 0.001), following a rather curvilinear trend (Figure 1).

**Table 2:** Linear regression results of AIS prevalence by the geographic latitude. The   inclusion of the (Latitude)^2^  term implies a possible quadratic   relationship.

**Number of obs=**	287939	**R-squared=**	0.509	
***F (*2,288036)=**	149257.39	**Adj R-squared=**	0.509	
**(Prob >*F*)**	<0.001	**Root MSE=**	0.7122	
**Prevalence**	**Unstandardized coefficient (B)**	**P > t**	**95% Conf.**	**Interval**
Latitude	0.024758	< 0.001	0.024045	0.0254713
Latitude^2	0.000484	<0.001	0.000472	0.0004965
Constant (Intercept)	0.712796	<0.001	0.703129	0.7224631

### The age at menarch

**Figure 2:**
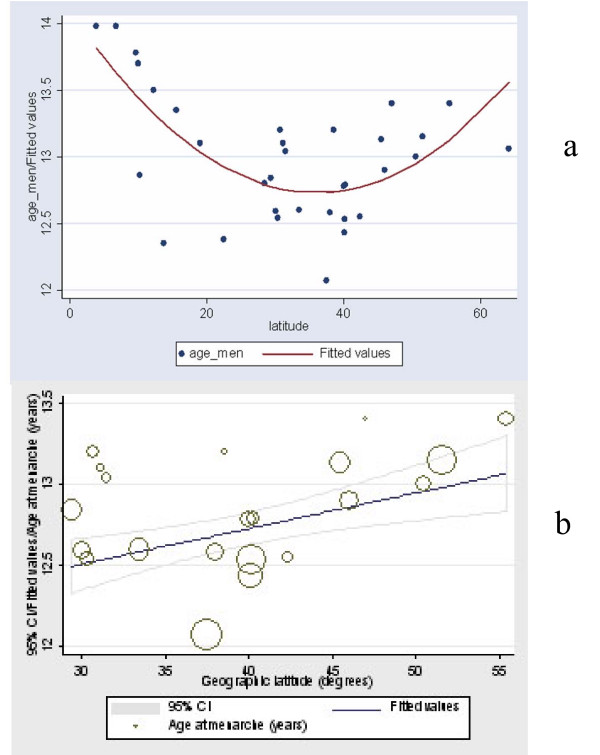
**A**. Illustration of the regression of age at menarche (in years) by latitude   (in degrees) (p < 0.001). **B**. The linear correlation between age at menarche   and geographic latitude is better shown in the figure where all observations   below latitude of 25 degrees are not described.

Age at menarche shows a decreasing trend as the geographic latitude approaches approximately the 25–30 degrees and then increases again toward 0 degrees (near the equator) (Table 3, Figure 2a).

**Table 3:** Demonstrates the city or geographical periphery where each study was performed, the geographic latitude in degrees, the age at menarche in years, and the author and year of publication.

**City or Geographical Periphery**	**Geographic Latitude in degrees**	**Age at menarche in years**	**Author and year of publication**
Oslo (Norway)	59,93	**16,00**	Rosenberg 1991 [21]
Copenhagen (Denmark)	55,43	**13,40**	Helm P & Grolund L 1998 [22]
Rotterdam (Netherlands)	51,55	**13,15**	Mul et al 2001 [23]
Brussels (Belgium)	50,51	**13,00**	Vercauteren M & Susanne C 1984 [24]
Switzerland	47,00	**13,40**	Largo RH & Prader A 1983 [25]
Pecs (Hungary)	46,00	**12,90**	Dober I & Kiralyfalvi L 1993 [26]
Zagreb (Croatia)	45,48	**13,13**	Prebeg Z & Bralic I 2000 [27]
L' Aquila (Italy)	42,36	**12,55**	Danubio et al 2004 [28]
Madrid (Spain)	40,26	**12,79**	Marroban MD, Mesa MS 2000 [29]
Coimbra (Portugal)	40,15	**12,53**	Padez C & Rocha MA 2003 [30]
Dayton (Ohio, USA)	40,13	**12,43**	Chumlea et al 2003 [31]
Sardinia (Italy)	40,00	**12,78**	Floris G et al 1987 [32]
Athens (Greece)	38,00	**12,58**	Dacou-Voutetakis et al 1983 [33]
Thriasion Pedion, (Greece)	37,50	**12,07**	Grivas et al 2002 [34]
Columbia (USA)	38,53	**13,20**	Vadocz EA et al 2002 [35]
Atlanta (Georgia, USA)	33,46	**12,60**	Freedman et al 2002 [36]
Marrakech (Morocco)	31,49	**13,04**	Montero P et al 1999 [37]
Texas (USA)	31,14	**13,10**	Malina RM et al 1994 [38]
Chandigarh (India)	30,72	**13,20**	Sharma K 1990 [39]
Patiala (India)	30,35	**12,54**	Singh SP & Malhotra P 1988 [40]
Cairo (Egypt)	30,05	**12,59**	Attallah NL 1978 [41]
Shiraz (Iran)	29,38	**12,84**	Ayatollahi SM et al 2002 [42]
Zhejiang (China)	28,45	**12,80**	Hesketh T et al 2002 [43]
Hong Kong (China)	22,45	**12,38**	Huen KF et al 1997 [44]
Dominican Republic	19,00	**13,10**	Mancebo P et al 1990 [45]
Khartoum (Sudan)	15,55	**13,35**	Attallah NL et al 1983 [46]
Bangkok (Thailand)	13,73	**12,35**	Chompootaweep S et al 1997 [47]
Hausa (Nigeria)	12,24	**13,50**	Rehan N 1994 [48]
Carabobo (Venezuela)	10,23	**12,86**	Farid-Coupal N et al 1981 [49]
Nigeria	10,00	**13,70**	Oduntan SO et al 1976 [50]
Jaffna (Sri Lanka)	9,66	**13,78**	Prakash S & Pathmanathan G 1984 [51]
Kumasi (Ghana)	6,75	**13,98**	Adadevoh SW et al 1989 [52]
Yaunde (Cameroon)	3,85	**13,98**	Pasquet P et al 1999 [53]

The linear correlation between age at menarche and geographic latitude is better shown when all observations of lower latitude than 25 degrees are not described, (Figure 2b).

The age at menarche in healthy population regressed by latitude showed that there is a statistical significant correlation, (p < 0.001) (Table 4).

**Table 4:** Linear regression results of age at menarche by the geographic latitude.

**Number of obs=**	68763	**R-squared=**	0.342	
***F (*2,288036)=**	17855.41	**Adj R-squared=**	0.342	
**(Prob >*F*)**	<0.001	**Root MSE=**	0.409	
**Age at menarche**	**Unstandardized coefficient (B)**	**P > t**	**95% Conf.**	**Interval**
Latitude	-0.1123787	<0.001	-0.1135448	-0.1112126
Latitude^2	0.0018579	<0.001	0.0018381	0.0018776
Constant (Intercept)	14.07617	<0.001	14.06203	14.09031

## Discussion

The regression curves of AIS prevalence and age at menarche by latitude are following a parallel decrement, especially in latitudes northern than 30°, as it is shown in figures 1 and 2 respectively.

### The prevalence of AIS

On reviewing the literature, prevalence figures quoted by various authors, serve to emphasize an apparent divergence in different parts of the world. The figures may be reflecting differences in the definition of a scoliotic curve, the methods of clinical examination, the thresholds for referral, the age group screened and as to whether the studies are based on random sampling or a longitudinal survey of individual children over some years. On the other hand recorded figures may represent real environmental, geographical, genetic or racial influences [54].

In the present study the AIS prevalence decreases as the geographic latitude approaches the equator. The parallel decrement of their regression curves in northern hemisphere implicates the role of numerous factors related to latitude in the pathogenesis of AIS.

### Geographic latitude and age at menarche

Although the age at menarche is to some extent influenced by family heredity, body weight, photic input and season, it seems more susceptible to modification by certain socioeconomic level and by specific disorders (such as diabetes, obesity and blindness) [55].

The influence of the geography should be distinguished between the effects of actual geographic factors (latitude, longitude, altitude, humidity and lighting) and those of the socioeconomic circumstances [55].

According to the critical weight hypothesis [56], there is a cut-off level for Body Mass Index (BMI) in relation to pubertal development. Beyond such degree of weight or BMI, there is no influence on age at menarche. The improved nutritional status among black girls in the recent years is implicated for an 8-month decrease of the median menarcheal age in black girls in a 20-year period, in contrast to a smaller decrease of 2 months in white girls at Bogalusa, a semi rural community near New Orleans, in the United States [36]. A stabilization of a previously decreasing trend of menarcheal age was recorded in studies from Norway [57] and from the Netherlands, [23] signifying the role of the nutritional status in sexual maturation.

Racial differences may also exist in other characteristics that have been suggested to influence pubertal development, such as the secretion of hormones by the hypothalamus, anterior pituitary, and ovary [58] or the social stress [59]. Recent studies on leptin, a protein which appears at higher levels among black girls [60] have suggested that it could act as a link between fat tissue and the central activation of the hypothalamus [61].

Earlier reports have not supported the belief once widely held, that sexual development occurs at an earlier age in the tropics than in temperate zones. It was reported that climate in itself has little or no effect on menarche [55,62].

In countries with geographic latitude less than 30° there is different climate. In this region, the so called climatologically moderately favourable belt, the sunshine is estimated at 2 500 h/year [63].

In this area apart from the different climate, there is a low socioeconomic status that critically influences the anthropometric dimensions of adolescents. Poverty and children malnutrition results in delay of skeletal maturation. This delay reflects a situation in which the environmental conditions, in terms of nutrition, do not allow the child to reach the optimal genetic potential. Thus the reported age at menarche from those countries is rather confusing. Additionally, there are no reports on AIS prevalence from those countries. A possible association between age at menarche and AIS prevalence in geographic latitudes less than 30° is therefore not realistic.

### Light and age at menarche

The effect of light on human biology is an issue that has rather not received much attention.

Environmental lighting exerts important effects on the age at which sexual maturation occurs in birds, [64,65] and in monestrous mammals and polyestrous rodents [66].

A retinal response to environmental lighting mediates an expanding list of neuroendocrine effects, including control of pubescence, ovulation, and a large number of daily rhythms [67,68].

The pattern of decrease of age at menarche with the shown specific trend as the geographic latitude approaches approximately down to 30 northern degrees could be attributed to daylight and cloudiness duration. The amount of sunlight and the quality of light (degrading relative irradiance and wavelength) may play a major role for the different initiation of menses in above-mentioned latitudes. It is useful for our study to cite the world distribution of solar radiation.

### World distribution of solar radiation and quality of light [63]

Solar radiation is unevenly distributed throughout the world because of such variables as solar altitude, which is associated with latitude and season, and atmospheric conditions, which are determined by cloud coverage and degree of pollution. The reported guidelines for the broad identification of the geographic areas with favourable solar energy conditions in the Northern Hemisphere based on the collection of the direct component of sunlight are described below. Similar conditions apply for the Southern Hemisphere [69].

The most favourable belt is (15–35°N). *It has over 3000 h/year of sunshine and limited cloud coverage. *More than 90% of the incident solar radiation comes as direct radiation.

The moderately favourable belt (0–15°N), or equatorial belt, has high atmospheric humidity and cloudiness that tend to increase the proportion of the scattered radiation. The global solar intensity is almost uniform throughout the year as the seasonal variations are only slight. *Sunshine is estimated at 2 500 h/year*.

In the less favourable belt (35–45°N), the scattering of the solar radiation is significantly increased because of the higher latitudes and lower solar altitude. In addition, cloudiness and atmospheric pollution are important factors that tend to reduce sharply the solar radiation intensity. However, regions beyond 45°N have less favourable conditions for the use of direct solar radiation. This is because almost half of it is in the form of scattered radiation, which is more difficult to collect for use [63]. As it is also reported the ultraviolet irradiance is different in different latitudes, and a regression model of the form: lnE = a_0 _+ a_1_cosΘ + a_2_Z, (where E is the seasonally integrated irradiance, Θ is the latitude and Z is the elevation of the observing site above sea level) explains 98.4% of the variance in the specific data sets of the related report [70]. The variation in human illumination exposure at different latitudes has also been elsewhere reported [71].

Moreover the different quality of light (wavelength) is probably responsible for the later age at menarche presented by girls living in higher altitudes, as it is reported in the available literature [39,72], affecting probably the melatonin rhythm. This issue is in accordance with the reported increase in 6-Hydroxymelatonin excretion in humans during ascent to high altitudes [73]

### Melatonin and age at menarche

At the onset of puberty, the hypothalamus, after being quiescent, resumes a marked pulsatile secretion of Gonadotrophine Releasing Hormone (GnRH), leading to an increased secretion of pituitary gonadotropines (Luteinizing Hormone [LH], and Follicle Stimulating Hormone [FSH]), especially during night, which in turn stimulates the gonadal functions. Cerebral adrenergic and/or dopamine neurotransmitters, endogenous opioids, and melatonin from the pineal gland are some of the neuroendocrine factors thought to be involved in the onset of puberty [74]

**Figure 3:**
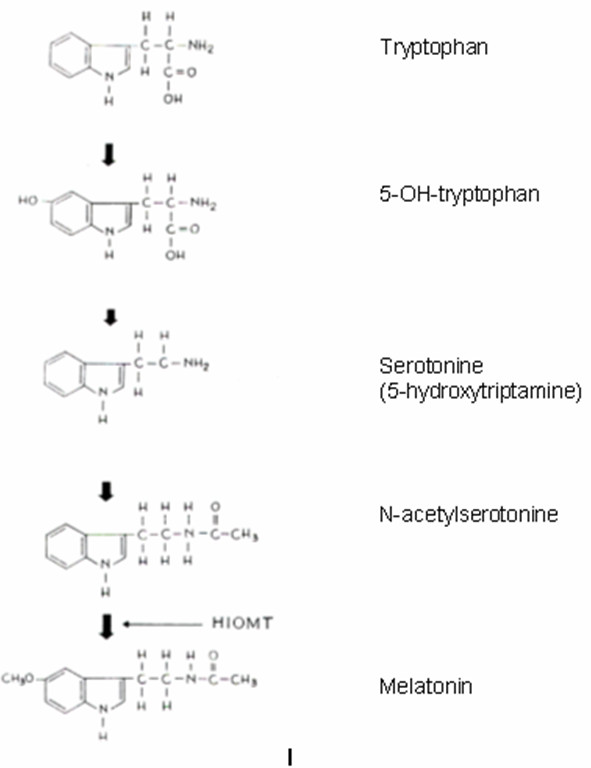
Biosynthesis of melatonin.

Melatonin is a hormone derived from the amino acid tryptophan and is secreted by the pineal gland. The enzyme Hydroxy-Indolo-O-Methyl-Transferase (HIOMT) converts the N-acetyl-serotonin to melatonin (Figure 3).

Melatonin production is stimulated by darkness. The lack of light on the retina, through the optic nerve and the pre-ganglionic sympathetic fibres of the upper cervical ganglion of the sympathetic trunk reaches firstly the centre of vision and then through the post-ganglionic fibres reaches the pineal gland and provokes the release of norepinephrine from the end plates of the sympathetic system. Norepinephrine mediates the entrance of tryptophan into the pineal gland and controls the activity of many enzymes and mainly that of HIOMT, which is important for the synthesis of melatonin [75]. The pineal gland concentration of HIOMT reduces during day time and increases during night time. Thus the diurnal variation of melatonin is due to environmental light conditions. Consequently darkness leads to melatonin over production and light reduces the melatonin production [76,77]. Melatonin is the main mediator that transfers the changes of the environmental light to the human cells [78].

Melatonin acts on the gonads indirectly, reducing the secretion of gonadotropines and mainly that of LH. This finding may explains the inhibition of ovulation in the Eskimos during the months of winter night period, and the increased rates of melatonin in normal women during night- time.

A controversy exists whether blind girls with no light stimuli experience delayed puberty. Zacharias and Wurtman in 1964 reported earlier age at menarche of girls suffering retroretineal fibroplasia than normal girls [77], while Thomas and Pizzarello in 1967 reported no difference [79] and Segos in 1995 reported that blind girls present late age at menarche [80] Jafarey et al in 1970 and 1971 reported that artificial lighting results in decreasing of age at menarche [81,82]. A more thorough research is required in this issue.

Mean night time serum melatonin concentration presents an increased peak value at 1–3 yr of age (329.5 ± 42.0 pg/mL), and lessens thereafter, averaging 62.5 ± 9.0 pg/mL in individuals aged 15–20 yr and 29.2 ± 6.1 pg/mL in old age (70–90 yr) [83] The decrease in nocturnal serum melatonin in children and adolescents correlated with body weight and body surface area, whereas no such correlation was found at a later age [83].

There are numerous limitations to interpret studies of melatonin in human subjects because of methodological considerations, such as the use of single blood samples collected during the day or the night, failure to include age related characteristics of melatonin secretion, lack of control of the actual duration and intensity of light exposure, and use of broad clinical features without hormonal markers to define puberty [84].

Sexual maturation can be delayed in experimental animals by exogenous melatonin administration or by short day exposure [85,88]. A rapid decrease in melatonin has also been observed during successful treatment of patients with delayed puberty [85].

### AIS and age at menarche

In this study, two linear regression procedures conducted, one between the prevalence of idiopathic scoliosis and the geographic latitude, and the other between the age at menarche and the geographic latitude. The reader could ask the reasonable question why a direct correlation between the prevalence of idiopathic scoliosis and the age at menarche was not performed? The answer is that due to the study design, the data were collected from two different groups of publications, the first consisting of publications regarding the prevalence of scoliosis whereas the second describing the different age at menarche in various geographic latitudes. As a result, the statistical analysis was done with two different samples. Since the data for the prevalence of IS and the age at menarche referred to different latitudes, a direct correlation could not be carried out. Moreover, no articles which had data for age at menarche and the prevalence of IS for a specific latitude could be found. At long last the results of this study suggest a possible correlation between age at menarche and prevalence of IS based on statistical analysis.

AIS is associated with pubertal growth spurt and its progression decelerates after completion of skeletal maturity. The age at onset of menarche is indicative of the remaining growth potential of girls. Late onset of menses correlates with delayed skeletal maturity and it implies that there is a potential for progression of a scoliotic curve. In a scoliotic girl, it is very important to predict the evolution of the curve and to advise accordingly the patient and her family. Goldberg et al [86,87] have shown that menarcheal status is a more meaningful predictor of curve stability than Risser sign, because at menarche peak growth velocity is already past [88] and although Risser stage 1 is still, on average, 8 months away [89,90], the possibility for more growth and significant progression, especially for smaller curves is declining rapidly. Furthermore, girls who are pre-menarcheal at diagnosis have a higher prevalence of surgery, as the menarcheal status alone will divide a female patient group into those who are at significant risk of surgery and those who are not [91]. A clear association exists between the deterioration of a scoliotic curve and periods of rapid growth, such those occurring before pubarche.

### Melatonin and AIS

The role of melatonin deficiency in AIS pathogenesis has been proposed by Machida et al [92] who produced scoliosis similar to those of human adolescent idiopathic scoliosis in pinealectomized chickens. When pinealectomized chickens were administered melatonin, the scoliosis was prevented. They proposed that a defect in melatonin production might be related to the aetiology of human idiopathic scoliosis.

There is a controversy whether lower animal models are appropriate for studying scoliosis. Chickens present extrapineal sites of melatonin production [93,94] that contribute to circulating melatonin levels, in contrast to humans that no extrapineal sources affect the circadian rhythm of melatonin [95]. There is a loss of the nighttime melatonin peak and a drop in basal levels below detection after pinealectomy in humans [96,97], whereas in chickens secretion is preserved with elimination of the nighttime peak [98]. Melatonin's actions appear to differ between humans, other mammals, and other vertebrates [93,95-97,99]. In humans melatonin may act by modulating calcium-activated calmodulin [100].

In a prospective study on pinealectomy in bipedal nonhuman primates, Cheung et al reported that none of the 18 monkeys developed scoliosis in a mean follow up period of 28 months. This study strongly suggests that the possible etiologic factors producing idiopathic scoliosis in lower animals are different from primates, and findings in lower animals cannot necessarily be extrapolated to human beings [101].

Studies on melatonin circadian secretion in humans report contradictory results. Machida et al reported that the integrated melatonin concentration throughout the 24-hour period in the patients who had a progressive curve was significantly lower than the level in the patients who had a stable curve or in the control group [102].

Hilibrand et al in a study that was conducted to test the suggestion of Machida found, in contrary to their research hypothesis, that morning urine melatonin levels were higher in patients with scoliosis than in the control subjects, but the differences between these values were not statistically significant [103]. They also reported a non-statistically significant difference of morning urine melatonin levels between patients with progressive and stable curves. The authors concluded that there was no difference in melatonin levels, as reflected in morning and evening urine collections, between patients with AIS and control subjects.

Fegan et al, in a case-control study of 24-hour urinary melatonin production in patients with adolescent idiopathic scoliosis reported that in adolescent idiopathic scoliosis, neither the presentation with a stable spinal deformity, nor presentation with a severe deformity requiring surgery is associated with melatonin deficiency [104].

No difference was also found by Bagnall et al in single day- and night-time measurements of the serum melatonin level in a control group and 7 patients with progressive adolescent idiopathic scoliosis [105]. In addition, no difference was found by Brodner et al in the urinary excretion of 6-sufatoxyl-melatonin in a control group and patients with progressive adolescent idiopathic scoliosis [106].

The argument against the alleged role of melatonin deficiency in AIS pathogenesis is contained in reports highlighting that an increased incidence of scoliosis has not been observed in children after pinealectomy or pineal irradiation because of pineal neoplasias, although they have a lack of serum melatonin [107-109].

### Geographic latitude, lifestyle differences and scoliosis

The different prevalence of IS by latitude could be probably attributed also to different lifestyle of people at deferent geographical latitudes. The seating people in the northern hemisphere societies are reducing their lumbar lordosis contrary to the physiologic sagittal profile exhibited in photographs of the African people of the tribe Nuba, as seen in the book of German photographer Leni Riefenstahl. Reduced lumbar lordosis (the seating effect in the northern hemisphere societies) frequently occurs in correlation with the lateral spinal curvature. Loss of lumbar lordosis seems to be a fundamental problem leading to a destabilization of the spine also in frontal and coronal plane and vice versa correction of lumbar lordosis seems to correct spinal deformities also in fontal and coronal plane [110]. This mechanistic approach to scoliotic deformity prevalence which is quite different to the biological approach discussed in this study probably needs to be addressed with pertinent research in human groups living in different latitudes, which is lacking so far from the available literature.

### A hypothesis

It has been assumed that there are two types of pathogenetic factors for AIS, the initiating and those that cause progression. Initiating factors that can meaningfully be distinguished from progressing factors would eventually faint or disappear, while progressive factors, which are generally thought to be a mechanical process, are associating with curve magnitude [111].

A possible preservation of high levels of melatonin secretion during the pre-menarcheal period in scoliotic girls due to light insufficiency in northern countries is associated with delay of the age at menarche. These high levels of melatonin are possibly identifiable before presentation of AIS, but would not be apparent at the time of clinical presentation of AIS in the vast majority of cases. The pre-menarcheal elevated levels of melatonin could be considered as a possible initiating factor of idiopathic scoliosis and it does not correlate with the severity and the site of the curve. It alters growth by lengthening the period of spine vulnerability while other pre-existing or aetiological factors are contributing to the development of AIS. Longitudinal studies on melatonin secretion in pre-pubertal girls that are at risk to develop AIS (i.e. with trunk asymmetry but no radiographic evidence of AIS) could be undertaken in order to test this hypothesis.

The clinical relevance and therapeutic implication that could also be derived from this study is that in northern latitudes, in girls with anticipated progressive scoliosis with no menarche, hormonal treatment in order to commence it might be of potential value to stop progression.

## Conclusion

In this survey it appears that late age at menarche is parallel with higher prevalence of AIS, especially in latitudes northern than 30 degrees. Pubarche appears later in girls that live in northern latitudes and thus prolongs the period of spine vulnerability while other pre-existing or aetiological factors are contributing to the development of AIS.

## Abbreviations

Adolescent Idiopathic Scoliosis (AIS)

Hydroxy-Indolo-O-Methyl-Transferase (HIOMT)

Gonadotrophine Releasing Hormone (GnRH),

Luteinizing Hormone (LH),

Follicle Stimulating Hormone (FSH)

## Authors' contributions

T BG conceived the idea of the presented study, was responsible for the methodological setting of the study, performed the major part of literature review and has written the manuscript. EV contributed in the statistical analysis of the study, performed part of literature review and also contributed by reviewing, text editing and adding certain parts of the manuscript. VM performed part of literature review, documented the latitude of the centre where a particular retrieved from Medline paper was originated. CM contributed in the statistical analysis of the study. GK Performed a part of literature review. All authors have read and approved the final manuscript.
